# Unemployment in chronic airflow obstruction around the world: results from the BOLD study

**DOI:** 10.1183/13993003.00499-2017

**Published:** 2017-09-21

**Authors:** Rune Grønseth, Marta Erdal, Wan C. Tan, Daniel O. Obaseki, Andre F.S. Amaral, Thorarinn Gislason, Sanjay K. Juvekar, Parvaiz A. Koul, Michael Studnicka, Sundeep Salvi, Peter Burney, A. Sonia Buist, William M. Vollmer, Ane Johannessen

**Affiliations:** 1 Dept of Thoracic Medicine, Haukeland University Hospital, Bergen, Norway; 2 Dept of Clinical Science, University of Bergen, Bergen, Norway; 3 UBC James Hogg Research Centre, St Paul's Hospital, Vancouver, BC, Canada; 4 Dept of Medicine, Obafemi Awolowo University, Ile-Ife, Nigeria; 5 National Heart and Lung Institute, Imperial College London, London, UK; 6 Dept of Respiratory Medicine and Sleep, Faculty of Medicine, University of Iceland, Landspitali University Hospital, Reykjavik, Iceland; 7 Vadu Health and Demographic Surveillance System, KEM Hospital Research Centre Pune, Pune, India; 8 Dept of Internal and Pulmonary Medicine, SheriKashmir Institute of Medical Sciences, Srinagar, India; 9 Dept of Pulmonary Medicine, Paracelsus Medical University, Salzburg, Austria; 10 Chest Research Foundation, Chest Research Foundation, Pune, India; 11 Pulmonary and Critical Care Medicine, UHN67, Oregon Health and Science University, Portland, OR, USA; 12 Kaiser Permanente Center for Health Research, Portland, OR, USA; 13 Centre for International Health, Dept of Global Public Health and Primary Care, University of Bergen, Bergen, Norway

## Abstract

We aimed to examine associations between chronic airflow obstruction (CAO) and unemployment across the world.

Cross-sectional data from 26 sites in the Burden of Obstructive Lung Disease (BOLD) study were used to analyse effects of CAO on unemployment. Odds ratios for unemployment in subjects aged 40–65 years were estimated using a multilevel mixed-effects generalised linear model with study site as random effect. Site-by-site heterogeneity was assessed using individual participant data meta-analyses.

Out of 18 710 participants, 11.3% had CAO. The ratio of unemployed subjects with CAO divided by subjects without CAO showed large site discrepancies, although these were no longer significant after adjusting for age, sex, smoking and education. The site-adjusted odds ratio (95% CI) for unemployment was 1.79 (1.41–2.27) for CAO cases, decreasing to 1.43 (1.14–1.79) after adjusting for sociodemographic factors, comorbidities and forced vital capacity. Of other covariates that were associated with unemployment, age and education were important risk factors in high-income sites (4.02 (3.53–4.57) and 3.86 (2.80–5.30), respectively), while female sex was important in low- to middle-income sites (3.23 (2.66–3.91)).

In the global BOLD study, CAO was associated with increased levels of unemployment, even after adjusting for sociodemographic factors, comorbidities and lung function.

## Abstract 2

**Chronic airflow obstruction increases risk of unemployment, and is a burden to welfare systems worldwide** http://ow.ly/cxzv30cQ17A

## Introduction

Chronic airflow obstruction (CAO) is the primary characteristic of patients with chronic obstructive pulmonary disease (COPD) and affects up to one in five adults, depending on where they live, according to data from the Burden of Obstructive Lung Disease (BOLD) study [1]. COPD is expected to keep its position as the third most important cause of death worldwide [2], and imposes a substantial burden on quality of life [3] and healthcare utilisation [4]. So far, data on productivity-related burden of CAO or COPD have been scant [4].

Only three population-based studies have provided employment rates in CAO [5–7]. Erdal *et al*. [5] showed that 55% of individuals with CAO from a general Norwegian population were in a paid job, *versus* 87% of controls without CAO. However, controls were younger and had higher levels of education and the authors did not examine employment in multivariate analyses. Jansson *et al*. [6] examined CAO-specific disability in northern Sweden, but did not include a control group and did not report employment rates. In the PLATINO (Latin American Project for Research in Pulmonary Obstruction) study undertaken in five Latin-American countries, Montes de Oca *et al*. [7] showed that the workforce participation among subjects with CAO was lower than in healthy subjects (41.8% *versus* 57.1%). However, in multivariable analyses they found that higher age, dyspnoea, number of comorbid conditions, female sex and lower education were associated with unemployment, whereas CAO was only of borderline significance.

The BOLD study is a large international study providing population-based estimates of the prevalence and burden of CAO. One of the primary objectives of the BOLD study is to estimate disease burden in terms of activity limitation and economic impact [8]. In the current analysis, we have compared estimates of employment status in BOLD participants with and without CAO across the world.

## Methods

The BOLD protocol has been published previously [8]. It was written in compliance with the Helsinki declaration and is approved by local ethics committees at all sites. All participants provided written consent.

### Population

All participating sites were recruited from well-defined administrative areas with the goal of providing representative samples of the local population of ≥600 non-institutionalised persons aged ≥40 years. The current report includes participants from 26 sites (online supplementary material). Out of 22 118 participants providing interview data, 18 710 performed acceptable post-bronchodilator spirometry and were included in the descriptive part of the current analysis. However, when analysing risk for unemployment as outcome, all subjects aged ≥65 years (defined here as retirees) and homemakers/caregivers were excluded. After excluding these subjects, there were no CAO cases left in Tirana (Albania), so this centre is not part of the analyses assessing the effect of CAO on unemployment. Online supplementary table S1 lists sampling strategy and response rates for all sites.

### Data collection

The BOLD study is a cross-sectional study based on a structured, face-to-face interview using standardised questionnaires and pre-/post-bronchodilator spirometry. All study coworkers were trained and certified by BOLD coordinating centres.

The interviews gathered information on smoking habits, education, job status, self-reported comorbidities (hypertension, heart disease, diabetes, stroke and lung cancer) and respiratory symptoms (dyspnoea, wheezing and chronic bronchitis).

Participants indicated whether they had worked for income at any time in the preceding year or if they served as full-time homemakers/caregivers during that time frame. Since retirement was not formally captured under occupation, we excluded anyone aged ≥65 years from analyses involving employment status. All other individuals not being categorised as working, homemakers/caregivers or retirees were defined as unemployed. The main outcome for the current study was a dichotomous employment status where retirees (≥65 years) and homemakers/caregivers were excluded.

Never-smokers were individuals who had smoked <20 packs of cigarettes during their lifetime, or less than one cigarette daily for a year. Ex-smokers were those who reported an age at which they had stopped smoking. Education was categorised according to the highest level of completed schooling and divided into no schooling, primary school, middle school, high school, some college and completed college/university.

Dyspnoea was defined using the modified Medical Research Council (mMRC) questions (grades 0–4, see online supplementary material for details) [9]. Subjects reporting being unable to walk for reasons other than breathing problems were excluded from the dyspnoea variable. Wheezing was defined as attacks of wheezing associated with dyspnoea in the past 12 months. Chronic bronchitis was defined as productive cough on most days in 3 months per year for at least two consecutive years.

Post-bronchodilator spirometry was performed using a hand-held spirometer (EasyOne; ndd Medizintechnik, Zürich, Switzerland) according to American Thoracic Society standards [10], before and ≥15 min after inhalation of 200 μg salbutamol through a large-volume spacer. For quality control, all individual manoeuvers were reviewed by a pulmonary function reading centre.

Predicted values of forced expiratory volume in 1 s (FEV_1_), forced vital capacity (FVC) and FEV_1_/FVC ratio were estimated from equations for caucasians from the third National Health and Nutrition Examination Survey (NHANES-III) [11]. Spirometric CAO was defined as post-bronchodilator FEV_1_/FVC below lower limit of normal (LLN).

### Analysis

The sample size of the BOLD study was set to be able to provide robust CAO prevalence estimates at the individual sites [8]. No power calculations were performed *a priori* for employment status.

For unadjusted comparisons of individuals with and without CAO, we used Pearson Chi-squared (categorical variables) and t-tests (continuous variables). To illustrate differences in unemployment and CAO in different parts of the world, we stratified descriptive analyses by high-income sites (Sydney (Australia), Salzburg (Austria), Vancouver (Canada), London (UK), Tartu (Estonia), Hannover (Germany), Reykjavik (Iceland), Maastricht (the Netherlands), Bergen (Norway), Krakow (Poland), Lisbon (Portugal), Uppsala (Sweden), Lexington (KY, USA)) and low- to middle-income sites (Guangzhou (China), Mumbai (India), Pune (India), Manila (Philippines), Nampicuan Talugtug (Philippines), Annaba (Algeria), Cape Town (South Africa), Adana (Turkey), Kashmir (India), Sousse (Tunisia), Ile-Ife (Nigeria) and Fes (Morocco)). Income categories were based on the gross national income per capita (GNIPC) of the country, with the cut point between low-to-middle income and high income being GNIPC 10 000 US$. [12]. We also calculated a risk ratio for unemployment associated with CAO as the prevalence of unemployment in CAO subjects divided by the prevalence of unemployment in non-CAO subjects, using a log-binomial generalised linear model (in Stata (StataCorp, College Station, TX, USA) specified as *glm* with *fam(bin)* and *link(log)*). A risk ratio >1 indicates higher risk of unemployment associated with CAO, while a ratio <1 indicates lower risk of unemployment associated with CAO. To illustrate sex differences in CAO status across sites, we tabulated study sites and CAO status, stratified by sex.

Multivariable analyses for the pooled dataset were conducted using a multilevel mixed-effects generalised linear model (online supplementary material). An alternative approach would be a fixed-effect model. The difference to the chosen mixed-effects approach would be that the latter treats the sites as a random sample of all possible sites, whereas the former would tend to focus more exclusively on the sites that were included in the study. The BOLD sites are in some sense a random sample of broader sites to which we wish to make an inference.

The main predictor variable was spirometric CAO. We fitted five mixed models, all adjusting for site as a cluster level variable. We first identified the total effect of CAO on unemployment in a model with no additional covariates included (model 1). Model 2 added demographic variables (age, sex and education) and smoking habits. Model 2 was extended into model 3 by adding comorbidities. Model 4 included FVC in addition to model 3 covariates. As our multivariable analyses include height, age and sex, which are the main components when using % predicted values, we thus chose to analyse lung function in terms of absolute values. In addition, a recent publication from the European Community Respiratory Health Survey III study has indicated that FVC in absolute values (lung size) is able to explain most of the difference in symptom burden between males and females [13]. FVC is a robust indicator of lung disease, especially when obstruction is already taken into account. In addition, we included model 5 with respiratory symptoms in addition to the model 4 covariates (online supplementary material). Details of comorbidities and symptoms are presented in the online supplementary material. Covariates added in each model were added not as independent risk factors for unemployment, but as potential confounding or mediating factors influencing the effect of CAO on unemployment. In addition, models 2–5 were performed separately for high-income sites and for low- to middle-income sites.

In individual participant data meta-analyses, we estimated site-specific and overall odds ratios for CAO on unemployment in forest plots, with increasing adjustment corresponding to models 1–5 (except for the site adjustment). The Stata command used was *ipdmetan* which performs a two-stage individual participant data meta-analysis using the inverse-variance method. Unlike traditional meta-analysis, the individual participant data meta-analysis in *ipdmetan* fits a specified model to the data of one site at a time, making use of all individual participants within the sites. The two-stage approach derives aggregate data in each site separately and then combines these in a traditional meta-analysis model. The I^2^ statistic was reported to display the percentage of total variation across sites which was due to true site-by-site heterogeneity rather than what would be expected by chance alone (see the online supplementary material for more details) [14].

All analyses were performed using Stata SE version 14 for Macintosh OSX.

## Results

Out of 18 710 participants, 2123 (11.3%) had CAO. Compared to subjects without CAO, those with CAO were older, had lower education levels, more smoking exposure, more comorbidities, more respiratory symptoms and substantially lower FEV_1_ (table 1). Overall, CAO was more common in males than in females. However, site-specific prevalence estimates stratified by sex showed that for some centres the sex ratio was reversed (online supplementary table S2). Excluding those aged ≥65 years, 36.7% of participants with CAO reported paid work during the preceding year, whereas 53.2% of participants without CAO had undertaken paid work during the preceding year.

**TABLE 1:** Study participant characteristics in the Burden of Obstructive Lung Disease (BOLD) study by chronic airflow obstruction (CAO)

	**Spirometric CAO**	**No spirometric** **CAO**	**Total**
**Subjects**	2123	16 587	18 710
**Female**	46.4 (44.2–48.5)	51.9 (51.1–52.6)	51.3 (50.5–52.0)
**Age years**	60.7±11.9	55.2±11.0	55.8±11.3
**Smoking**			
Never-smoker	33.9 (32.0–36.0)	57.2 (56.4–58.0)	54.6 (53.8–55.3)
Ex-smoker	31.0 (29.1–33.0)	23.9 (23.2–24.5)	24.7 (24.1–25.3)
Current smoker	35.1 (33.1–37.1)	18.9 (18.4–19.5)	20.8 (20.2–21.4)
**Education**			
None	14.7 (13. 2–16.3)	12.1 (11.6–12.6)	12.4 (11.9–12.9)
Primary school	21.7 (20.0–23.5)	15.7 (15.2–16.3)	16.4 (15.9–16.9)
Middle school	17.0 (15.5–18.7)	16.0 (15.5–16.6)	16.1 (15.6–16.7)
High school	24.7 (22.9–26.6)	26.2 (25.5–26.8)	26.0 (25.4–26.6)
Some college	11.1 (9.8–12.5)	12.8 (12.3–13.4)	12.6 (12.2–13.1)
College/university	10.9 (9.6–12.3)	17.2 (16.6–17.8)	16.5 (15.9–17.0)
**Job status**			
Paid work	36.7 (34.7–38.8)	53.2 (52.4–53.9)	51.3 (50.6–52.0)
Homemaker/caregiver	14.8 (13.3–16.4)	13.5 (13.0–14.0)	13.7 (13.2–14.1)
Unemployed	19.6 (18.0–21.4)	16.2 (15.6–16.8)	16.6 (16.1–17.1)
Above retirement age	28.9 (27.0–30.8)	17.1 (16.6–17.7)	18.5 (17.9–19.0)
**Lung function**			
FVC % pred	89.2±21.8	90.3±16.1	90.2±16.9
FEV_1_ % pred	69.2±21.4	92.0±16.7	89.4±18.7
**Self-reported doctor's diagnosis**			
COPD	15.3 (13.8–16.9)	2.4 (2.2–2.6)	3.9 (3.6–4.2)
Hypertension	32.9 (30.9–34.9)	26.2 (25.6–26.9)	27.0 (26.3–27.6)
Heart disease	14.3 (12.9–15.9)	10.0 (9.5–10.4)	10.5 (10.0–10.9)
Diabetes	7.2 (6.1–8.3)	7.5 (7.1–7.9)	7.5 (7.1–7.9)
Stroke	3.1 (2.4–3.9)	1.9 (1.7–2.1)	2.0 (1.8–2.2)
Lung cancer	0.7 (0.4– 1.1)	0.2 (0.1– 0.3)	0.3 (0.2– 0.3)
**Dyspnoea**			
mMRC 0	55.8 (53.5–58.0)	78.8 (78.1–79.4)	76.2 (75.6–76.9)
mMRC 1	17.1 (15.5–18.9)	12.1 (11.6–12.6)	12.7 (12.2–13.2)
mMRC 2	13.2 (11.7–14.8)	5.6 (5.2–5.9)	6.4 (6.1–6.8)
mMRC 3	8.5 (7.3–9.8)	2.7 (2.5–3.0)	3.3 (3.1–3.6)
mMRC 4	5.5 (4.5–6.6)	0.9 (0.7–1.0)	1.4 (1.2–1.6)
**Attack of wheezing in past 12 months**	22.3 (20.6–24.1)	6.3 (5.9–6.6)	8.1 (7.7–8.5)
**Chronic bronchitis**	15.4 (13.9–17.0)	5.1 (4.8–5.5)	6.3 (5.9–6.6)

Data are presented as n, % (95% CI) or mean±sd. n=18 710 subjects from 26 study sites. All comparisons between CAO and non-CAO were significant (p<0.01, Pearson Chi-squared test for categorical variables, t-test for continuous variables) except for self-reported diabetes. Missing data: smoking habits n=11 (n=1 CAO, n=10 non-CAO); education n=25 (n=4 CAO, n=21 non-CAO); hypertension, diabetes, stroke, lung cancer and heart disease n=1 (n=1 CAO); dyspnoea n=1834 (n=258 CAO, n=1576 non-CAO), mostly because of other reasons for having trouble walking; wheezing n=11 (n=2 CAO, n=9 non-CAO); chronic bronchitis n=0. FVC: forced vital capacity; FEV_1_: forced expiratory volume in 1 s; COPD: chronic obstructive pulmonary disease; mMRC: modified Medical Research Council scale.

**FIGURE 1:**
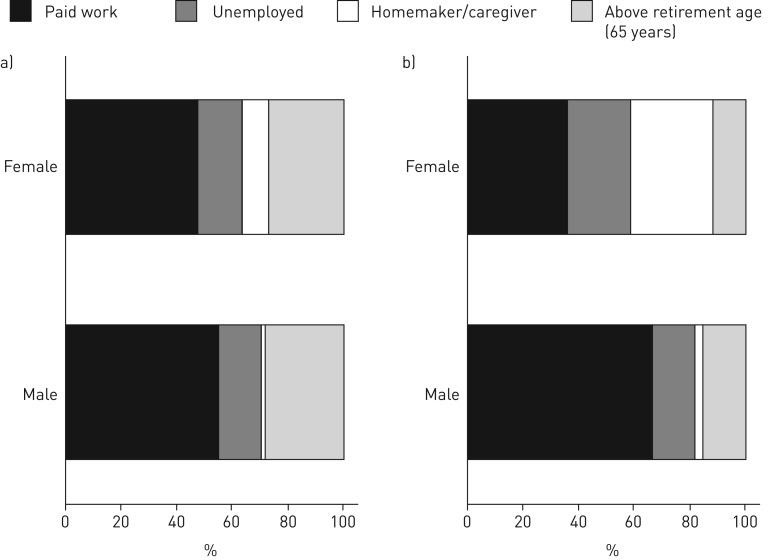
Distribution of job status by sex for participants in a) high-, and b) low- to middle-income countries. n=18 710.

Figure 1 shows that more males than females reported current paid employment in both high- and low- to middle-income countries, but the difference was larger in low- to middle-income countries. This appeared to be explained by a substantial proportion of female unpaid homemakers/caregivers in low- to middle-income countries. More details on sex differences in employment status are given in online supplementary table S3.

Table 2 shows unemployment by CAO status in each study site, excluding homemakers, caregivers and retirees (subjects aged ≥65 years). Despite a wide variation in unemployment rates by site, there was a fairly consistent pattern of higher unemployment among individuals with CAO in high-income sites. This pattern was less clear in the low- to middle-income sites.

**TABLE 2:** Unemployment rates: prevalence of unemployment by site and spirometric chronic airflow obstruction (CAO) status

	**Subjects^#^** **n**	**Unemployment %**		**Crude OR (95% CI)^¶^**
		**CAO**	**No CAO**	
**Total**	11 675			
**High-income**				
Bergen, Norway	397	20.0	9.5	2.1 (1.0–4.2)
Hannover, Germany	361	25.0	20.8	1.2 (0.6–2.5)
Krakow, Poland	350	57.9	41.4	1.4 (1.0–1.9)
Lexington, USA	305	61.0	27.7	2.2 (1.6–3.0)
Lisbon, Portugal	320	53.9	39.8	1.4 (0.9–2.0)
London, UK	427	40.4	24.3	1.7 (1.1–2.4)
Maastricht, the Netherlands	396	31.3	20.4	1.5 (1.0–2.3)
Reykjavik, Iceland	557	14.0	3.3	4.2 (1.8–10.1)
Salzburg, Austria	860	35.2	25.4	1.4 (1.1–1.8)
Sydney, Australia	339	20.0	15.3	1.3 (0.6–3.0)
Tartu, Estonia	348	20.0	7.8	2.6 (0.9–7.5)
Uppsala, Sweden	371	23.8	6.0	4.0 (1.7–9.5)
Vancouver, Canada	594	21.8	11.5	1.9 (1.1–3.3)
**Low- to middle-income**				
Adana, Turkey	487	41.1	45.4	0.9 (0.7–1.2)
Annaba, Algeria	408	50.0	24.6	2.0 (1.2–3.3)
Cape Town, South Africa	510	52.2	33.5	1.6 (1.2–2.0)
Fes, Morocco	335	41.7	53.7	0.8 (0.5–1.3)
Guangzhou, China	359	35.7	49.9	0.7 (0.4–1.5)
Ile-Ife, Nigeria	667	5.1	7.6	0.7 (0.2–2.7)
Kashmir, India	366	7.6	1.3	5.8 (1.5–22.9)
Manila, Philippines	594	10.3	19.5	0.5 (0.2–1.4)
Mumbai, India	250	17.7	10.3	1.7 (0.6–5.1)
Nampicuan Talugtug, Philippines	493	23.2	14.7	1.6 (0.9–2.7)
Pune, India	671	6.5	4.1	1.6 (0.4–6.4)
Sousse, Tunisia	390	53.3	46.1	1.2 (0.7–1.9)
Tirana, Albania	520	0.0	5.0	

^#^: retirees (age limit defined as ≥65 years) and homemakers/caregivers were excluded from the analysis. ^¶^: calculated based on prevalence of unemployment among subjects with CAO divided by prevalence of unemployment among subjects without CAO. A ratio >1 indicates higher unemployment prevalence among CAO subjects than among non-CAO subjects, while a ratio <1 indicates lower unemployment prevalence among CAO subjects.

In multivariable analyses, we assessed the odds ratio of being unemployed by CAO status and an increasing number of covariates (table 3). The first model showed that when we adjusted for site, the odds ratio (95% CI) of being unemployed was 1.79 (1.41–2.27) for participants with CAO. Adding the traditional confounders sex, age, smoking habits and education in model 2 decreased the odds ratio for unemployment in participants with CAO (OR reduction from 1.79 to 1.44), but the effect remained statistically significant. Further addition of comorbidities (model 3) and FVC (model 4) had little effect on the association between unemployment and CAO, even when these variables themselves were significantly associated with unemployment: the presence of comorbidities and declining FVC were all associated with increased odds of being unemployed. Table 3 shows that excess unemployment among those with CAO is partially explained by sex, age, smoking and education, but not explained additionally by comorbidities and FVC. When respiratory symptoms were added (online supplementary table S4), these were also significantly associated with unemployment and appeared to explain some of the effects of CAO. In this model, the odds ratio for CAO independent of reported symptoms fell to 1.26 (95% CI 1.00–1.57). Substituting self-reported COPD for LLN-defined CAO in our analyses increased the odds ratio of not being in paid work from 1.43 (95% CI 1.14–1.79) to 3.31 (95% CI 2.17–5.05) (additional analysis, data not shown). However, while the prevalence of spirometry-defined CAO was 11.3% in BOLD, the prevalence of self-reported COPD was only 1.2%, and while 36.7% of the spirometry-defined participants with CAO were in paid employment, the corresponding figure for the self-reported COPD cases was only 25% (results not shown).

**TABLE 3:** OR (95% CI) for unemployment for lower limit of normal-defined chronic airflow obstruction (CAO) and other risk factors, with an increasing degree of adjustment (demographic characteristics, comorbidities and forced vital capacity (FVC))

	**Model 1**	**Model 2**	**Model 3**	**Model 4**
**Spirometric CAO**	1.79 (1.41–2.27)	1.44 (1.15–1.81)	1.45 (1.15–1.82)	1.43 (1.14–1.79)
**FVC 10 percentage points decrease in % pred**				1.08 (1.04–1.12)
**Female**		2.07 (1.85–2.32)	2.10 (1.87–2.36)	2.10 (1.87–2.35)
**Age 10-year increment**		3.09 (2.85–3.35)	2.91 (2.68–3.15)	2.90 (2.67–3.15)
**Smoking status**				
Current smoker		0.96 (0.83–1.10)	0.98 (0.85–1.13)	0.98 (0.85–1.13)
Ex-smoker		1.15 (1.01–1.32)	1.13 (0.99–1.30)	1.14 (0.99–1.30)
**Education**				
Some college		1.51 (1.23–1.85)	1.49 (1.22–1.84)	1.49 (1.21–1.83)
High school		2.03 (1.71–2.42)	2.02 (1.69–2.41)	2.01 (1.68–2.39)
Middle school		2.24 (1.83–2.73)	2.20 (1.80–2.69)	2.18 (1.79–2.67)
Primary school		2.78 (2.27–3.41)	2.76 (2.25–3.39)	2.72 (2.22–3.35)
No education		2.73 (2.09–3.57)	2.69 (2.05–3.51)	2.66 (2.03–3.49)
**Comorbidities**				
Hypertension			1.29 (1.13–1.46)	1.26 (1.10–1.43)
Heart disease			1.54 (1.27–1.86)	1.51 (1.25–1.83)
Diabetes			1.51 (1.23–1.85)	1.47 (1.19–1.80)
Stroke			1.82 (1.16–2.86)	1.80 (1.15–2.83)
Lung cancer			2.34 (0.81–6.76)	2.38 (0.82–6.93)

Adjustment variables: no fixed effects (model 1); age, sex, education and smoking (model 2); model 2 adjustment + comorbidities (model 3); model 3 adjustment + FVC (model 4). All five models were fit using a multilevel mixed-effects generalised linear model with study site included as random effect to account for within-site clustering. Reference values for categorical variables: no CAO, male, never-smoker, university education, no hypertension, no heart disease, no diabetes, no stroke and no lung cancer. n=11 675. Retirees (age limit defined as ≥65 years) and homemakers/caregivers were excluded from the analysis.

To examine how the observed associations varied by country income, we performed multivariable analyses separately for high-income and low- to middle-income sites (table 4). CAO was a significant risk factor for unemployment in all models in high-income sites, but not in low- to middle-income sites. While age and lower education level were important risk factors for unemployment in high-income sites, female sex was the most pronounced risk factor for unemployment in low- to middle-income sites. To further depict the sex variation in job status, we created online supplementary table S3, which shows the prevalence of job status categories among males and females in each site. This table illustrates that almost no sites had more females than males in paid work (with Lexington, Lisbon and Ile-Ife as the only three exceptions). Further on, focusing on the low- to middle-income sites, this table demonstrates that the difference in “unemployed” job status between the sexes were very high in some sites, with the mean difference being 46.1% more unemployed females than males. There were some sites that had more unemployed males than females, but these were few (Annaba, Cape Town, Kashmir, Mumbai and Pune), and the mean difference was low (5.5%). In model 5, dyspnoea was an additional important risk factor for unemployment in high-income sites, together with age and education (online supplementary table S4).

**TABLE 4:** OR (95% CI) for unemployment for chronic airflow obstruction (CAO) and other risk factors, stratified by country income category, with increasing degree of adjustment (demographic characteristics, comorbidities and forced vital capacity (FVC))

	**Model 2**		**Model 3**		**Model 4**	
	**High income**	**Low to middle income**	**High income**	**Low to middle income**	**High income**	**Low to middle income**
**Spirometric CAO**	1.71 (1.17–2.49)	1.16 (0.78–1.73)	1.63 (1.16–2.28)	1.18 (0.79–1.76)	1.68 (1.16–2.45)	1.15 (0.77–1.71)
**FVC 10 percentage points decrease in % pred**					1.09 (1.03–1.15)	1.08 (1.02–1.14)
**Female**	1.36 (1.16–1.59)	3.34 (2.76–4.04)	1.43 (1.23–1.68)	3.25 (2.68–3.94)	1.44 (1.23–1.68)	3.23 (2.66–3.91)
**Age 10-year increment**	4.28 (3.77–4.86)	2.31 (2.06–2.59)	4.04 (3.55–4.59)	2.21 (1.96–2.48)	4.02 (3.53–4.57)	2.20 (1.96–2.47)
**Smoking status**						
Current smoker	1.34 (1.09–1.65)	0.88 (0.72–1.09)	1.36 (1.11–1.68)	0.89 (0.72–1.10)	1.36 (1.10–1.67)	0.89 (0.72–1.10)
Ex-smoker	1.31 (1.09–1.56)	1.15 (0.90–1.47)	1.28 (1.07–1.53)	1.13 (0.88–1.44)	1.29 (1.08–1.54)	1.12 (0.88–1.43)
**Education**						
Some college	1.85 (1.45–2.38)	0.96 (0.60–1.52)	1.83 (1.43–2.36)	0.95 (0.60–1.52)	1.83 (1.42–2.35)	0.97 (0.61–1.54)
High school	2.30 (1.84–2.88)	1.26 (0.92–1.73)	2.27 (1.81–2.84)	1.28 (0.93–1.76)	2.24 (1.79–2.81)	1.30 (0.94–1.78)
Middle school	3.65 (2.73–4.87)	1.23 (0.89–1.70)	3.54 (2.64–4.74)	1.26 (0.91–1.74)	3.49 (2.60–4.67)	1.26 (0.91–1.75)
Primary school	4.14 (3.02–5.66)	1.52 (1.10–2.10)	3.90 (2.84–5.37)	1.56 (1.13–2.16)	3.86 (2.80–5.30)	1.56 (1.13–2.16)
No education	1.96 (0.61–6.32)	1.61 (1.13–2.30)	2.01 (0.62–6.43)	1.64 (1.15–2.34)	1.98 (0.61–6.43)	1.65 (1.15–2.35)
**Comorbidities**						
Hypertension			1.25 (1.05–1.49)	1.25 (1.02–1.53)	1.22 (1.02–1.45)	1.22 (0.99–1.50)
Heart disease			1.56 (1.22–2.00)	1.20 (0.86–1.67)	1.53 (1.19–1.96)	1.18 (0.85–1.64)
Diabetes			1.53 (1.14–2.04)	1.39 (1.01–1.92)	1.46 (1.09–1.95)	1.38 (1.00–1.91)
Stroke			2.17 (1.14–4.13)	1.58 (0.83–3.02)	2.15 (1.13–4.10)	1.56 (0.82–2.99)
Lung cancer			2.87 (0.89–9.30)	1.01 (0.04–28.54)	2.86 (0.89–9.26)	1.03 (0.04–28.37)

Adjustment variables: no fixed effects (model 1); age, sex, education and smoking (model 2); model 2 adjustment + comorbidities (model 3); model 3 adjustment + FVC (model 4). All five models were fit using a multilevel mixed-effects generalised linear model with study site included as random effect to account for within-site clustering. Separate analyses were performed for high-income countries and low- to middle-income countries. Reference values for categorical variables: no CAO, male, never-smoker, university education, no hypertension, no heart disease, no diabetes, no stroke and no lung cancer. n=11 675. Retirees (age limit defined as ≥65 years) and homemakers/caregivers were excluded from the analysis.

**FIGURE 2:**
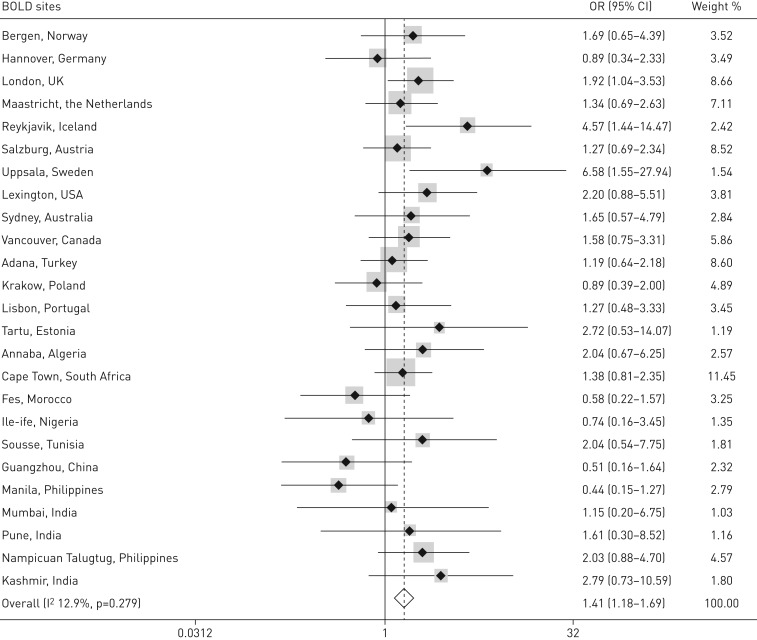
Odds ratios (95% CI) for unemployment for lower limit of normal-defined chronic airflow obstruction, adjusted for demographic characteristics, comorbidities and forced vital capacity (FVC). Adjustment variables: sex, age, smoking, education, hypertension, heart disease, diabetes, stroke, lung cancer and FVC. n=11 675, meta-analysis with results across sites and overall. Retirees (age limit defined as 65 years) and homemakers/caregivers excluded. BOLD: Burden of Obstructive Lung Disease study.

To present the association between CAO and unemployment by site, and to examine site heterogeneity, we performed individual participant data meta-analyses with forest plots of odds ratios and overall I^2^ statistics (figure 2 and online supplementary figures S1–S4). The overall odds ratio (95% CI) for unemployment among CAO subjects after adjusting for sex, age, smoking, education, comorbidities and FVC (*i.e.* the equivalent of model 4, but without site adjustment) was 1.41 (1.18–1.69) with site-by-site heterogeneity (I^2^) of 12.9% (p=0.279). Meta-analyses with covariates corresponding to models 1, 2, 3 and 5 are shown in online supplementary figures S1–S4, and show that there is no significant site heterogeneity in the association between airflow obstruction and unemployment when adjusting for the covariates in models 2, 3 and 5. However, in crude analysis (model 1), there is significant site heterogeneity (I^2^ 49.1%, p=0.003).

## Discussion

The unweighted prevalence of spirometry-defined CAO was 11.3% in this sample of almost 19 000 participants from the global BOLD study. The association between CAO and unemployment varied across sites in crude analyses, but the site heterogeneity lost significance after adjustment for relevant covariates: CAO was an overall important risk factor for unemployment after adjusting for sex, age, smoking, education, comorbidities and even FVC. When looking at high-income and low- to middle-income sites separately, this association was only statistically significant in high-income sites. Regarding other covariates, age and education were important risk factors for unemployment in high-income sites, while female sex was important for unemployment in low- to middle-income sites.

Comparable population-based studies have previously observed similar prevalence rates of COPD as the CAO rates found in the present study. The PLATINO (Latin-American Pulmonary Obstruction Investigation Project) study found the prevalence to be within the range of 7.8–19.7% [15], Hansen *et al.* [16] found the overall COPD prevalence in a Danish general population to be 12%, and the systematic review by Adeloye *et al.* [17] found the global prevalence of population-based spirometrically defined COPD to be 11.7%.

Only one multicentre study has previously provided population-based estimates of unemployment in CAO, identifying CAO using spirometry. In accordance with our findings, the PLATINO study, performed in five Latin-American countries, estimated that 41.8% of participants with CAO and 57.1% of those without CAO had a paid job the preceding year [7]. In the multivariable analysis of the PLATINO study they found a borderline lower probability of paid work (OR 0.83, 95% CI 0.69–1.00) for CAO patients, and, as in our study, they found significant effects of age, sex, education, dyspnoea and comorbidities. However, the PLATINO study researchers adjusted for dyspnoea in their main model, and this has probably reduced the effect of spirometry-defined CAO on the probability of having paid work. We observed the same pattern in our study; while CAO was significantly associated with unemployment in our main model with OR 1.43 (adjusting for sex, age, smoking, education, comorbidities and FVC), the odds ratio decreased to 1.26 (although still remaining significant, with 95% CI 1.00–1.57) after adding reported dyspnoea and other respiratory symptoms. In line with this, we speculate that symptoms and severity of CAO would probably explain the bulk of unemployment, and that it would be better to study these disease aspects than merely spirometry measurements. However, even after adjusting for mMRC, wheezing with dyspnoea and symptoms of chronic bronchitis in our study, the effect of spirometry-defined CAO on unemployment was still significant (model 5; online supplementary material). This suggests that there are properties other than the burden of current wheezing, dyspnoea and bronchitis that lead to unemployment, and adding objectively measured CAO identifies the magnitude of these. For instance, the patient might experience other symptoms (*e.g.* asthenia), be a frequent exacerbator or there might be some degree of reporting bias.

Other studies on workforce participation of CAO patients have been based on self-reported COPD diagnosis and not spirometry [18–22]. Studies of self-reported COPD observe stronger associations between the disease and participation in the workplace than the current study. This difference might be due to a bias towards more severely affected patients in studies based on self-reports [23]. Lamprecht *et al.* [24] showed that >80% of subjects with post-bronchodilator FEV_1_/FVC <LLN were undiagnosed, and that less severe airflow obstruction was an important predictor lack of diagnosis.

The inclusion of undiagnosed CAO patients by state-of-the-art spirometric case detection in representative population-based samples is the main strength of the current study. All epidemiological studies are subject to selection bias to some degree, and the use of representative samples and mostly high cooperation rates (over half >70%) reduce the likelihood of strong biases from selection. Furthermore, our main outcome is categorical and objective, and less prone to bias [25, 26] than reports of diagnoses, although some of the covariates may be more prone to recall bias. In addition, we have used post-bronchodilator measurements, in accordance with international guidelines, and we have a large sample size from a general global population with standardised data collection across sites. In addition, we have built regression models based on *a priori* hypotheses of associations, rather than including all variables that were significant in bivariable analyses or by an automated stepwise approach.

Some limitations deserve to be mentioned. First of all, the BOLD study is a cross-sectional study, and as such we cannot infer temporality and we have no direct evidence that the CAO was directly responsible for the unemployment. It is not unthinkable that some of the association between CAO and unemployment is a result of unemployed participants being more susceptible to the disease, even if we have adjusted for education, age and smoking habits. Economic hardship in the form of unemployment can worsen individual unhealthy behaviours including smoking [27]. Second, the employment question is based on any paid work in the past year, and does not differentiate between full-time and part-time work. In other words, subjects who have needed to reduce their work participation due to CAO from full-time to part-time will still be defined as in paid work in our analysis. This may lead to an underestimation of associations between CAO and employment. Being able to present absolute rates of disease-related unemployment standardised at the site population level would have been an advantage, but as our data did not include census information with age distribution details from each site this was not feasible. Future research should preferably include such data for this purpose. Furthermore, lack of a direct question on retirement means that we may have underestimated the problem of unemployment above 65 years of age. Our chosen cut-off of 65 years as retirement age may have affected results in both directions. Third, our spirometry-derived variables were calculated from the NHANES III reference equation for caucasians. This is relatively uncontroversial for measures of FEV_1_/FVC in the age group 40–65 years, as normal values are not strongly associated with ethnicity. However, overall, the prevalence of spirometry-defined CAO (FEV_1_/FVC <LLN) will be slightly lower with NHANES reference values than with the recently recommended Global Lung Function Initiative reference values [28]. The difference would not be large enough for us to expect substantial differences in the associations observed in the present study. If anything, a higher CAO prevalence would lead to larger effects of CAO on unemployment, including more individuals with less severe obstruction. The use of NHANES may be more controversial for the measures of FVC than for the ratio measures. In this case, we have used FVC as a continuous variable so that the “lower limit of normal” is not an issue, and, as we have allowed a separate baseline in each centre and as most centres are ethnically homogeneous, this should not present a problem [29, 30]. Since the main focus of the present study was on associations rather than prevalences, we chose to implement the same reference values for the whole study population. This may allow for possible factors that might have affected the lung function at a national level to become apparent, instead of being lost with the use of different reference equations at each site. Fourth, regarding study limitations, the registration of never-smokers may have been somewhat exaggerated if there were participants who started smoking recently before study inclusion, but who had not yet reached 20 lifetime packs of cigarettes. However, the risk of this would seem small given that the youngest participants included in the study are aged 40 years. Lastly, there might be a bias toward more females responding as unemployed in low- to middle-income sites due to cultural differences where females might not have formal employment, although they attend work and have an informal income. This information bias might make the sex difference in the risk of being unemployed somewhat higher than the actual risk in these sites, but unfortunately it is beyond the potential of our dataset to disentangle this possible female misclassification. Online supplementary table S3 shows that the differences between males and females applied to almost all sites.

The association between CAO and unemployment was significant in overall analyses, but in stratified analyses we observed that the association was probably driven by high-income sites. There may be several reasons for this. First, subjects in low- to middle-income countries may have more prevalent diseases than CAO that render them vulnerable to unemployment. Second, there may be more heterogeneity in low- to middle-income sites than in high-income sites. Our analyses showed consistent results across the high-income sites that seemed to be more homogeneous than the low- to middle-income sites, where CAO was a risk factor for unemployment in some sites and almost a protective factor against unemployment in other sites. The suspicion was further strengthened by crude meta-analysis, showing significant site heterogeneity in the univariate association between CAO and unemployment. However, when other covariates were accounted for, the site heterogeneity lost significance. Third, other factors may be more important than health factors for unemployment risk in low- to middle-income countries. We observed that female sex was an important risk factor for unemployment in these sites, while age and education were important for the high-income sites. Traditional male/female roles in low- to middle-income countries may affect work-life participation to such a degree that they blur the association between health-related factors and unemployment. Such large sex differences in work participation were illustrated in online supplementary table S3 in the present study. And last, but not least, our results may be an indication of how disease burden act differently in high-*versus* low- to middle-income sites, due to a strictly economic component. In high-income sites those most severely affected are given the possibility to be economically sustained by the corresponding social security systems, while in low- to middle-income sites such alternatives are few or nonexistent. While in high-income sites, the welfare system bears the economic burden of disease, in low- to middle-income sites the people affected both bear the personal and the economic burden of disease.

In conclusion, we have found that work-life participation of subjects with CAO is overall lower than work-life participation of subjects without CAO, and that CAO is associated with unemployment after adjusting for sex, age, smoking, education, comorbidities and even FVC. There was no significant heterogeneity between sites, although stratified analyses showed that CAO may be of greater importance for unemployment in high-income sites. Our study shows the risk of unemployment among people with this prevalent respiratory disease, and illustrates how CAO considerably impacts productivity and social security systems worldwide.

## Supplementary material

10.1183/13993003.00499-2017.Supp1 **Please note:** supplementary material is not edited by the Editorial Office, and is uploaded as it has been supplied by the author.
Supplementary material ERJ-00499-2017_Supplement
Figure S1. Crude odds ratios (OR) with 95% confidence intervals (95% CI) for unemployment for LLN-defined CAO. N #x003D; 11675* subjects, meta-analysis with results across sites and overall. Legend: *Retirees (age limit defined as 65 years old) and homemakers/caregivers excluded. Corresponds to model 1 in Table 4 (but without site adjustment). ERJ-00499-2017_Figure_S1
Figure S2. Odds ratios (OR) with 95% confidence intervals (95% CI) for unemployment for LLN-defined CAO, adjusted for demographic characteristics*. N = 11675** subjects, meta-analysis with results across sites and overall. Legend: *Adjustment variables: gender, age, smoking, education. Corresponds to model 2 in Table 4 (but without site adjustment). **Retirees (age limit defined as 65 years old) and homemakers/caregivers excluded. ERJ-00499-2017_Figure_S2
Figure S3. Odds ratios (OR) with 95% confidence intervals (95% CI) for unemployment for LLN-defined CAO, adjusted for demographic characteristics, and comorbidities*. N #x003D; 11675** subjects, meta-analysis with results across sites and overall. Legend: *Adjustment variables: gender, age, smoking, education, hypertension, heart disease, diabetes, stroke, lung cancer. Corresponds to model 3 in table 4 (but without site adjustments). **Retirees (age limit 65 years old) and homemakers/caregivers excluded. ERJ-00499-2017_Figure_S3
Figure S4. Odds ratios (OR) with 95% confidence intervals (95% CI) for unemployment for LLN-defined CAO, adjusted for demographic characteristics, comorbidities, FVC, and respiratory symptoms*. N #x003D; 11675** subjects, meta-analysis with results across sites and overall. Legend: *Adjustment variables: gender, age, smoking, education, hypertension, heart disease, diabetes, stroke, lung cancer, FVC, dyspnea, dyspnoea and wheezing, chronic bronchitis symptoms. Corresponds to model 5 in table S3 (but without site adjustment). **Retirees (age limit 65 years old) and homemakers/caregivers excluded. ERJ-00499-2017_Figure_S4

## Disclosures

10.1183/13993003.00499-2017.Supp2 P. Burney ERJ-00499-2017_Burney
M. Erdal ERJ-00499-2017_Erdal
R. Grønseth ERJ-00499-2017_Gronseth
W.C. Tan ERJ-00499-2017_Tan
W.M. Vollmer ERJ-00499-2017_Vollmer
