# Supplementary material for: Unemployment in chronic airflow obstruction around the world: results from the BOLD study
Source: Eur Respir J. 2017 Sep 21;50(3):1700499. doi: 10.1183/13993003.00499-2017 (PMC5898950; doi:10.1183/13993003.00499-2017)
Supplement: Supplementary file 1 [file ERJ-00499-2017_Supplement.pdf]

SUPPLEMENTAL MATERIAL AND TABLES

Unemployment in chronic airflow obstruction around the world: Results from the BOLD study

Running head: Unemployment in CAO

Writing group

Rune Grønseth, nielsenrune@me.com, Department of Thoracic Medicine, Haukeland University Hospital, Bergen, Norway
Marta Erdal, marta.erdal@k2.uib.no, Department of Thoracic Medicine, Haukeland University Hospital, Bergen, Norway
Wan C. Tan, wan.tan@hli.ubc.ca, UBC James Hogg Research Centre, St Paul's Hospital, Vancouver, British Columbia, Canada
Daniel O. Obaseki, danseki@yahoo.com, Department of Medicine, Obafemi Awolowo University, Ile-Ife, Nigeria
Andre F. S. Amaral, a.amaral@imperial.ac.uk, National Heart and Lung Institute, Imperial College London, London, United Kingdom
Thorarinn Gislason, thorarig@landspitali.is, Faculty of Medicine, University of Iceland, Dept. of Respiratory Medicine and Sleep, Landspitali University Hospital, Reykjavik, Iceland
Sanjay K. Juvekar, sanjay.juvekar@gmail.com, Vadu Health and Demographic Surveillance System, KEM Hospital Research Centre Pune, Pune, India
Parvaiz A. Koul, parvaizk@gmail.com, Department of Internal & Pulmonary Medicine, SheriKashmir Institute of Medical Sciences, Srinagar, India
Michael Studnicka, M.Studnicka@salk.at, Department of Pulmonary Medicine, Paracelsus Medical University, Salzburg, Austria
Sundeep Salvi, ssalvi@crfindia.com, Chest Research Foundation, Chest Research Foundation, Pune, India
Peter Burney, p.burney@imperial.ac.uk, National Heart and Lung Institute, Imperial College London, London, United Kingdom
A. Sonia Buist, buists@ohsu.edu, Oregon Health & Science University, Oregon Health & Sciences University, Portland, Oregon, United States
William M. Vollmer, william.vollmer@kpchr.org, Kaiser Permanente Center for Health Research, Portland, Oregon, United States
Ane Johannessen, ane.johannessen@uib.no, Centre for International Health, Department of Global Public Health and Primary Care, University of Bergen, Bergen, Norway.

Corresponding author:
Marta Erdal, marta.erdal@k2.uib.no, Department of Thoracic Medicine, Haukeland University Hospital, Bergen, Norway

ONLINE SUPPLEMENT MATERIAL

METHODS

The current report includes participants from 26 sites: Guangzhou (China), Manila (Philippines), Mumbai (India) Nampicuan Talugtug (Philippines), Pune (India), Kashmir (India), Annaba (Algeria), Cape-Town (South Africa), Fes (Morocco), Ife (Nigeria), Sousse (Tunisia), Adana (Turkey), Krakow (Poland), Lisbon (Portugal), Tartu (Estonia), Tirana (Albania), Bergen (Norway), Hannover (Germany), London (United Kingdom), Maastricht (The Netherlands), Reykjavik (Iceland), Salzburg (Austria), Uppsala (Sweden), Lexington (USA), Sydney (Australia), and Vancouver (Canada).

Dyspnea was defined using the modified Medical Research Council questions with the following categories: only breathless with strenuous exercise (grade 0); short of breath when hurrying on the level or walking up a slight hill (grade 1); walk slower than people of own age or have to stop for breath when walking on own pace at level ground (grade 2); stop for breath after 100 m or a few minutes on the level (grade 3) and too breathless to leave the house or breathless when dressing or undressing (grade 4).

Multivariable analyses for the pooled data-set were conducted using multilevel mixed-effects generalized linear model (*meglm* in Stata), assuming unstructured covariance matrix with a binomial distribution and a logit link function, with study site included as random effect and with estimations of odds ratios for unemployment.

Comorbidities included in model 3 were hypertension, heart disease, diabetes, stroke and lung cancer. Respiratory symptoms included in model 5 were modified MRC dyspnea, wheezing and dyspnea, and chronic bronchitis symptoms) in addition to the Model 4 covariates.

The individual participant data meta-analyses were performed using *ipdmetan* in Stata, with binomial distribution, logit link function and robust variance estimates. The I^2 statistic (range 0-100%) was reported to display the percentage of total variation across sites which was due to true site-by-site heterogeneity. I^2 is defined as 100% x (Q-df)/Q, with Q being the classical measure of heterogeneity (Cochran's Q). I^2 is a simple and easily interpretable expression of site differences. Zero% indicates no observed heterogeneity (beyond what would be expected by

chance), while <25%, 25 - 50% and > 75% is characterised as low, moderate and high heterogeneity, respectively (13).

Table S1. Participation in 26 sites of the BOLD study.

Site	Sampling design	N*	N1*	N2*	Response rate (%)	Cooperation rate (%)
Guangzhou, China	Stratified random sample	602	473	461	87	87
Adana, Turkey	Stratified cluster sample	875	806	806	82	85
Salzburg, Austria	Stratified random sample	1349	1258	1253	65	67
Capetown, South Africa	Cluster sample	896	847	844	63	68
Reykjavik, Iceland	Simple random sample	758	757	757	81	84
Hannover, Germany	Stratified random sample	713	683	680	59	61
Krakow, Poland	Stratified random sample	603	526	526	78	79
Bergen, Norway	Stratified random sample	707	658	658	68	71
Vancouver, Canada	Random digit dialling	856	827	827	26	51
Manila, Philippines	Stratified cluster sample	918	893	892	58	58
Lexington, USA	Random digit dialling	563	508	508	14	27
Sydney, Australia	Stratified random sample	585	541	541	25	33
London, England	Stratified random sample	697	677	675	17	37
Uppsala, Sweden	Stratified random sample	588	547	547	61	63
Mumbai, India	Stratified cluster sample	515	440	439	55	66
Lisbon, Portugal	Stratified cluster sample	745	714	710	10	27
Maastricht, The Netherlands	Stratified random sample	634	590	589	48	55
Nampicuan & Talugtug , Philippines	Stratified cluster sample	991	722	722	86	86
Tartu, Estonia	Stratified random sample	658	615	614	49	70
Pune, India	Simple random sample	1388	849	845	97	97
Sousse, Tunisia	Stratified cluster sample	717	661	660	90	92
Srinagar, India	Stratified cluster sample	953	763	741	86.9	88
Ife, Nigeria	Stratified cluster sample	1148	904	865	76	98
Fes, Morocco	Cluster sample	966	769	760	98	98
Tirana, Albania	Cluster sample	997	941	928	82	84
Annaba, Algeria	Stratified random sample	917	892	862	95	95

*N - Total number of responders: defined as participants who completed the core questionnaire and performed post-bronchodilator FEV1 and/or FVC; N1 – Participants with technically acceptable post-bronchodilator FEV1 and FVC; N2 – included in the current paper: participants with post-bronchodilator FEV1/FVC, age ≥ 40 years and core questionnaire information.

Response rates: total number of responders divided by the total number of eligible individuals contacted. **Cooperation rates:** total number of responders divided by the total number of responders plus active refusers.

Table S2. Prevalence of chronic airflow obstruction (CAO) in %, across 26 sites in the BOLD study, stratified by gender. N = 18 710 subjects.

Site	N	Male	Female
**HIGH INCOME**			
Bergen, Norway	658	15% (324)	10% (334)
Hannover, Germany	680	10% (347)	8% (333)
Krakow, Poland	526	15% (266)	12% (260)
Lexington, USA	508	14% (206)	16% (302)
Lisbon, Portugal	710	14% (331)	10% (379)
London, UK	675	16% (321)	16% (354)
Maastricht, Netherlands	589	19% (299)	17% (290)
Reykjavik, Iceland	757	9% (403)	13% (354)
Salzburg, Austria	1253	13% (683)	19% (570)
Sydney, Australia	541	8% (265)	14% (276)
Tartu, Estonia	614	9% (309)	5% (305)
Uppsala, Sweden	547	10% (283)	8% (264)
Vancouver, Canada	827	13% (344)	12% (483)
**LOW-TO-MIDDLE INCOME**			
Adana, Turkey	806	20% (389)	9% (417)
Annaba, Algerie	862	10% (429)	4% (433)
Cape Town, South Africa	844	24% (314)	16% (530)
Fes, Morocco	760	12% (350)	8% (410)
Guangzhou, China	461	9% (229)	6% (232)
Ile-Ife, Nigeria	865	7% (339)	7% (526)
Kashmir, India	741	18% (407)	15% (334)
Manila, Philippines	892	13% (378)	5% (514)
Mumbai, India	439	6% (275)	8% (164)
Nampicuan Talugtug, Philippines	722	16% (356)	12% (366)
Pune, India	845	6% (502)	7% (343)
Sousse, Tunisia	660	8% (309)	2% (351)
Tirana, Albania	928	13% (463)	4% (465)

Denominators for the percentages are shown in parenthesis.

Table S3. Prevalence of job status categories in % among men (blue) and women (red), across 26 sites in the BOLD study. N = 18 710 subjects.

Site	N	Paid work	Homemaker/caregiver	Unemployed	Above retirement age
**HIGH INCOME**					
Bergen, Norway	658	61.4 / 51.8	0.0 / 1.2	6.8 / 9.6	31.8 / 37.4
Hannover, Germany	680	50.1 / 46.6	0.3 / 10.2	19.0 / 16.2	30.6 / 27.0
Krakow, Poland	526	45.9 / 31.2	1.9 / 11.9	30.1 / 32.7	22.1 / 24.2
Lexington, USA	508	40.3 / 44.7	1.5 / 17.2	35.0 / 26.2	23.2 / 11.9
Lisbon, Portugal	710	29.6 / 30.9	0.6 / 3.2	19.9 / 22.7	49.9 / 43.2
London, UK	675	53.6 / 41.5	0.9 / 11.0	17.1 / 18.4	28.4 / 29.1
Maastricht, Netherlands	589	59.2 / 47.2	0.3 / 23.5	16.7 / 13.5	23.8 / 15.8
Reykjavik, Iceland	757	76.4 / 66.1	6.5 / 18.1	2.5 / 4.8	14.6 / 11.0
Salzburg, Austria	1253	55.0 / 47.0	0.2 / 8.1	18.3 / 21.0	26.5 / 23.9
Sydney, Australia	541	58.1 / 51.1	1.9 / 12.3	10.6 / 12.0	29.4 / 24.6
Tartu, Estonia	614	57.3 / 49.8	1.0 / 3.3	5.5 / 4.6	26.2 / 42.3
Uppsala, Sweden	547	68.2 / 66.3	0.4 / 1.5	5.3 / 8.0	26.1 / 24.2
Vancouver, Canada	827	72.4 / 57.4	0.3 / 8.3	9.0 / 11.2	18.3 / 23.1
**LOW-TO-MIDDLE INCOME**					
Adana, Turkey	806	60.2 / 15.8	0.8 / 19.7	20.6 / 49.2	18.4 / 15.3
Annaba, Algerie	862	59.9 / 14.8	7.5 / 76.2	19.1 / 6.7	13.5 / 2.3
Cape Town, South Africa	844	50.6 / 34.3	4.5 / 32.5	28.7 / 22.6	16.2 / 10.6
Fes, Morocco	760	45.4 / 8.1	1.4 / 41.0	33.2 / 40.2	20.0 / 10.7
Guangzhou, China	461	51.1 / 28.9	0.0 / 0.4	28.4 / 50.9	20.5 / 19.8
Ile-Ife, Nigeria	865	72.3 / 73.6	0.3 / 0.6	4.4 / 6.8	23.0 / 19.0
Kashmir, India	741	80.8 / 8.7	11.1 / 88.9	1.5 / 0.9	6.6 / 1.5
Manila, Philippines	892	71.7 / 48.6	3.7 / 26.3	11.6 / 14.2	13.0 / 10.9
Mumbai, India	439	78.6 / 5.5	1.8 / 91.5	8.4 / 2.4	11.2 / 0.6
Nampicuan Talugtug, Philippines	722	76.4 / 39.9	4.2 / 35.0	7.3 / 13.9	12.1 / 11.2
Pune, India	845	80.1 / 72.0	0.4 / 20.4	4.6 / 2.0	14.9 / 5.6
Sousse, Tunisia	660	62.1 / 22.8	0.3 / 1.1	25.9 / 65.0	11.7 / 11.1
Tirana, Albania	928	58.1 / 52.5	38.7 / 44.5	2.8 / 2.8	0.4 / 0.2

Table S4: Odds ratios (OR) with 95% confidence intervals (95%CI) for unemployment for LLN-defined CAO and other risk factors, with increasing degree of adjustment (demographic characteristics, comorbidities, FVC, and respiratory symptoms)*. Overall analyses and stratified by country income category. N = 11 675.**

	**All sites**	**High income sites**	**Low to middle income sites**
	**Model 5**	**Model 5**	**Model 5**
Spirometric CAO	1.26 (1.00, 1.57)	1.48 (0.99, 2.21)	1.04 (0.71, 1.52)
FVC, 10 percentage points decrease in %predicted	1.05 (1.01, 1.09)	1.04 (0.99, 1.10)	1.06 (1.00, 1.12)
Female gender	2.01 (1.79, 2.26)	1.35 (1.15, 1.58)	3.14 (2.58, 3.81)
Age, 10yrs increment	2.94 (2.71, 3.19)	4.09 (3.59, 4.65)	2.22 (1.98, 2.50)
Smoking Status			
Current-smoker	0.95 (0.83, 1.10)	1.31 (1.05, 1.62)	0.87 (0.70, 1.08)
Ex-smoker	1.12 (0.97, 1.28)	1.27 (1.06, 1.52)	1.09 (0.85, 1.40)
Education			
Some college	1.47 (1.20, 1.81)	1.77 (1.38, 2.28)	0.96 (0.60, 1.53)
High school	1.97 (1.65, 2.35)	2.20 (1.75, 2.76)	1.27 (0.92, 1.74)
Middle school	2.08 (1.70, 2.55)	3.13 (2.32, 4.21)	1.24 (0.89, 1.71)
Primary school	2.56 (2.08, 3.15)	3.58 (2.59, 4.94)	1.50 (1.08, 2.07)
No education	2.41 (1.84, 3.16)	2.06 (0.64, 6.62)	1.55 (1.08, 2.22)
Hypertension	1.23 (1.08, 1.39)	1.17 (0.98, 1.40)	1.21 (0.98, 1.48)
Heart disease	1.37 (1.13, 1.66)	1.36 (1.06, 1.75)	1.08 (0.77, 1.51)
Diabetes	1.41 (1.15, 1.74)	1.33 (0.99, 1.80)	1.36 (0.98, 1.88)
Stroke	1.81 (1.15, 2.85)	2.07 (1.08, 3.97)	1.64 (0.85, 3.15)
Lung cancer	2.29 (0.77, 6.86)	2.58 (0.76, 8.76)	0.97 (0.04, 24.97)
Dyspnea category			
mMRC grade 1	1.16 (0.99, 1.35)	1.29 (1.03, 1.61)	0.99 (0.78, 1.26)
mMRC grade 2	1.65 (1.34, 2.03)	2.61 (1.89, 3.59)	1.22 (0.91, 1.63)
mMRC grade 3	2.02 (1.52, 2.69)	5.05 (3.01, 8.47)	1.25 (0.86, 1.80)
mMRC grade 4	3.32 (2.14, 5.14)	6.76 (2.74, 16.68)	2.41 (1.41, 4.11)
Wheezing and dyspnea	1.05 (0.86, 1.28)	0.94 (0.71, 1.25)	1.27 (0.94, 1.73)
Chronic bronchitis symptoms	0.99 (0.79, 1.23)	1.03 (0.76, 1.39)	0.81 (0.56, 1.16)

*Adjustment variables: age, gender, education, smoking, comorbidities, FVC and respiratory symptoms.

All analyses were performed using multilevel mixed-effects generalized linear model with study site included as random effect to account for within site clustering. Reference values for categorical variables: no CAO, males, never-smokers, university education, no hypertension, no heart disease, no diabetes, no stroke, no lung cancer, no dyspnea, no wheezing and dyspnea, no chronic bronchitis symptoms.

**Retirees (age limit defined as 65 years old) and homemakers/caregivers were excluded from the analysis.
