# Supplementary figures and images for: Unemployment in chronic airflow obstruction around the world: results from the BOLD study
Source: Eur Respir J. 2017 Sep 21;50(3):1700499. doi: 10.1183/13993003.00499-2017 (PMC5898950; doi:10.1183/13993003.00499-2017)

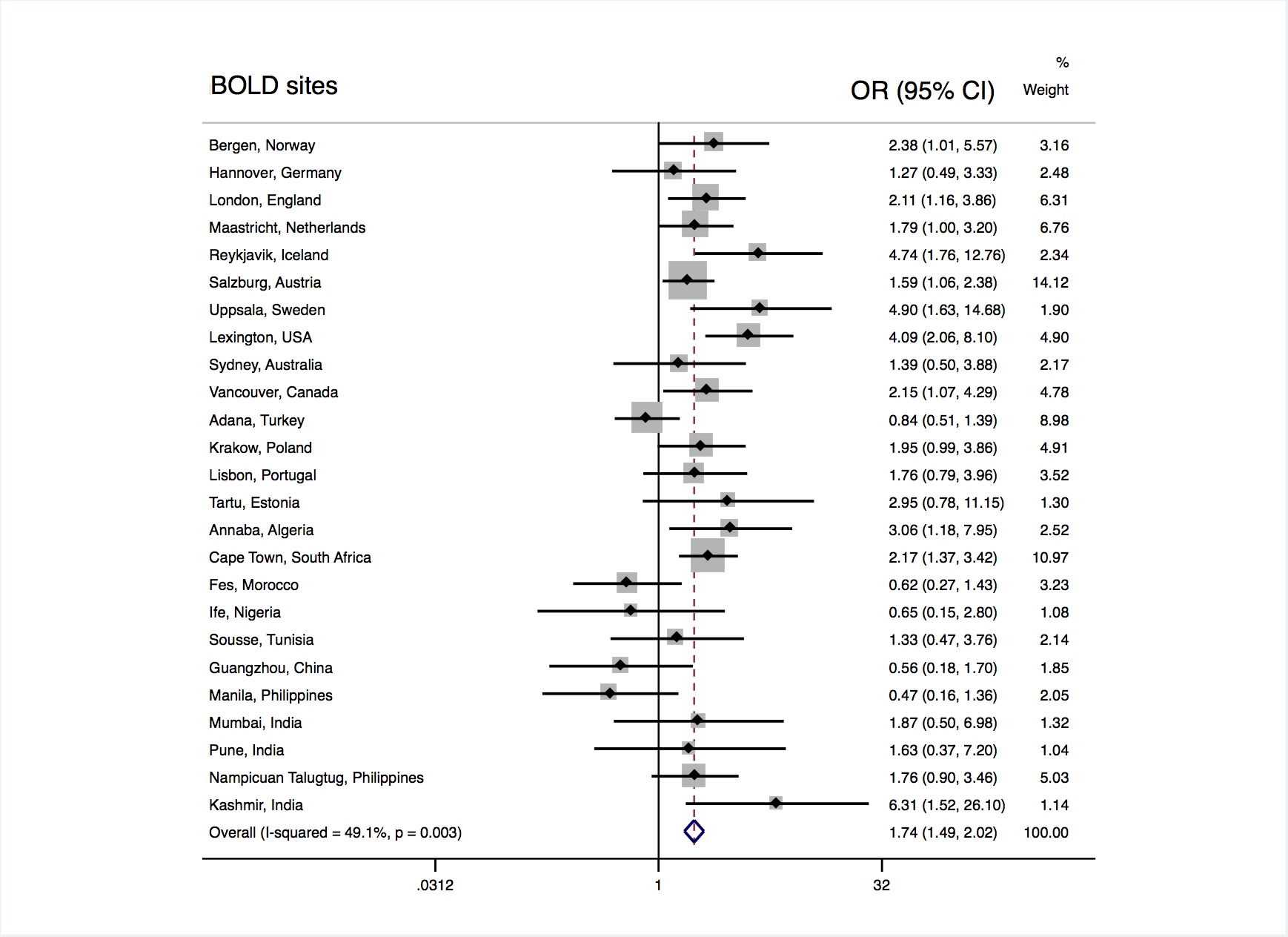

Supplement: Supplementary file 2 [file ERJ-00499-2017_Figure_S1.jpg]

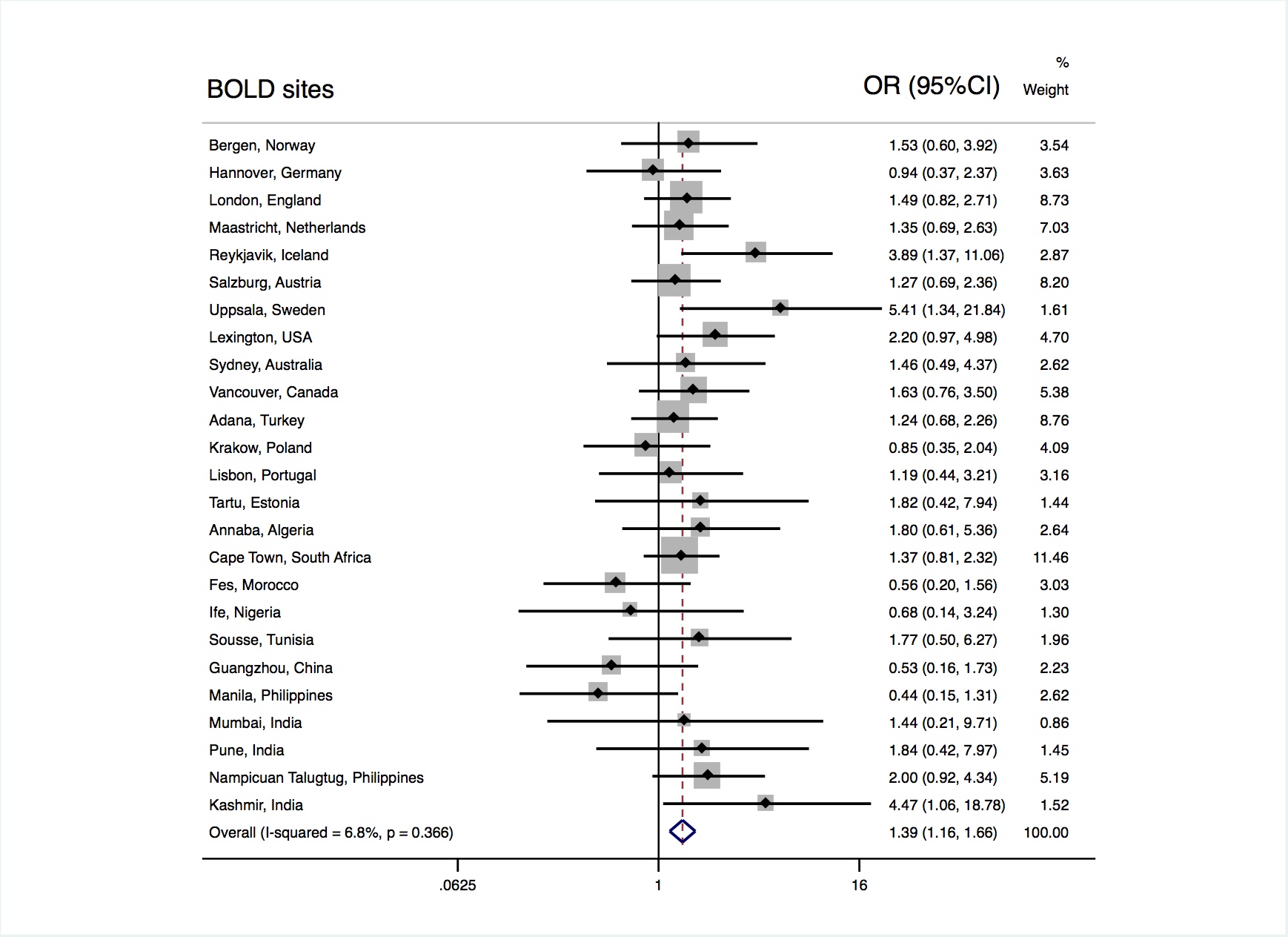

Supplement: Supplementary file 3 [file ERJ-00499-2017_Figure_S2.jpg]

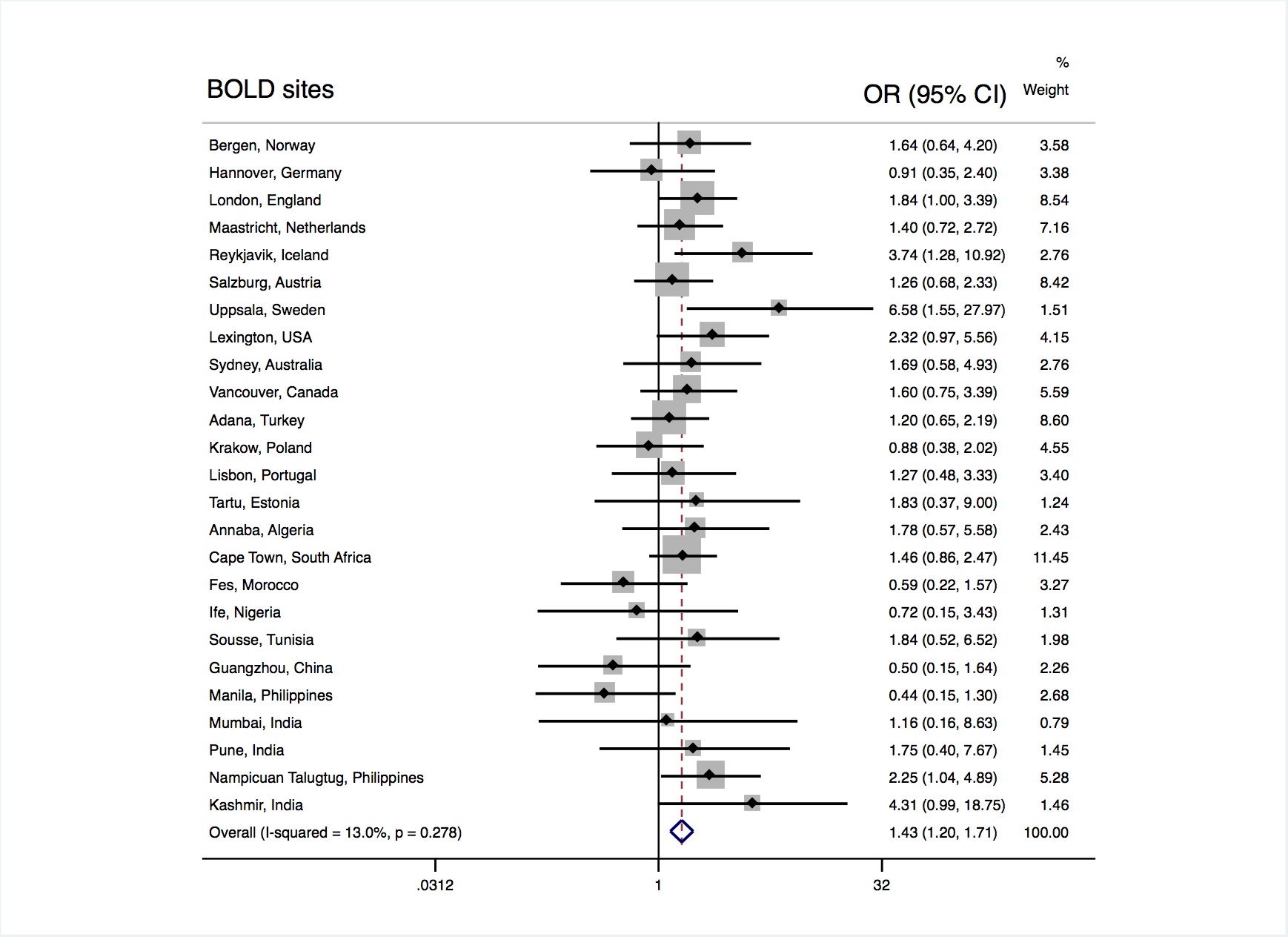

Supplement: Supplementary file 4 [file ERJ-00499-2017_Figure_S3.jpg]

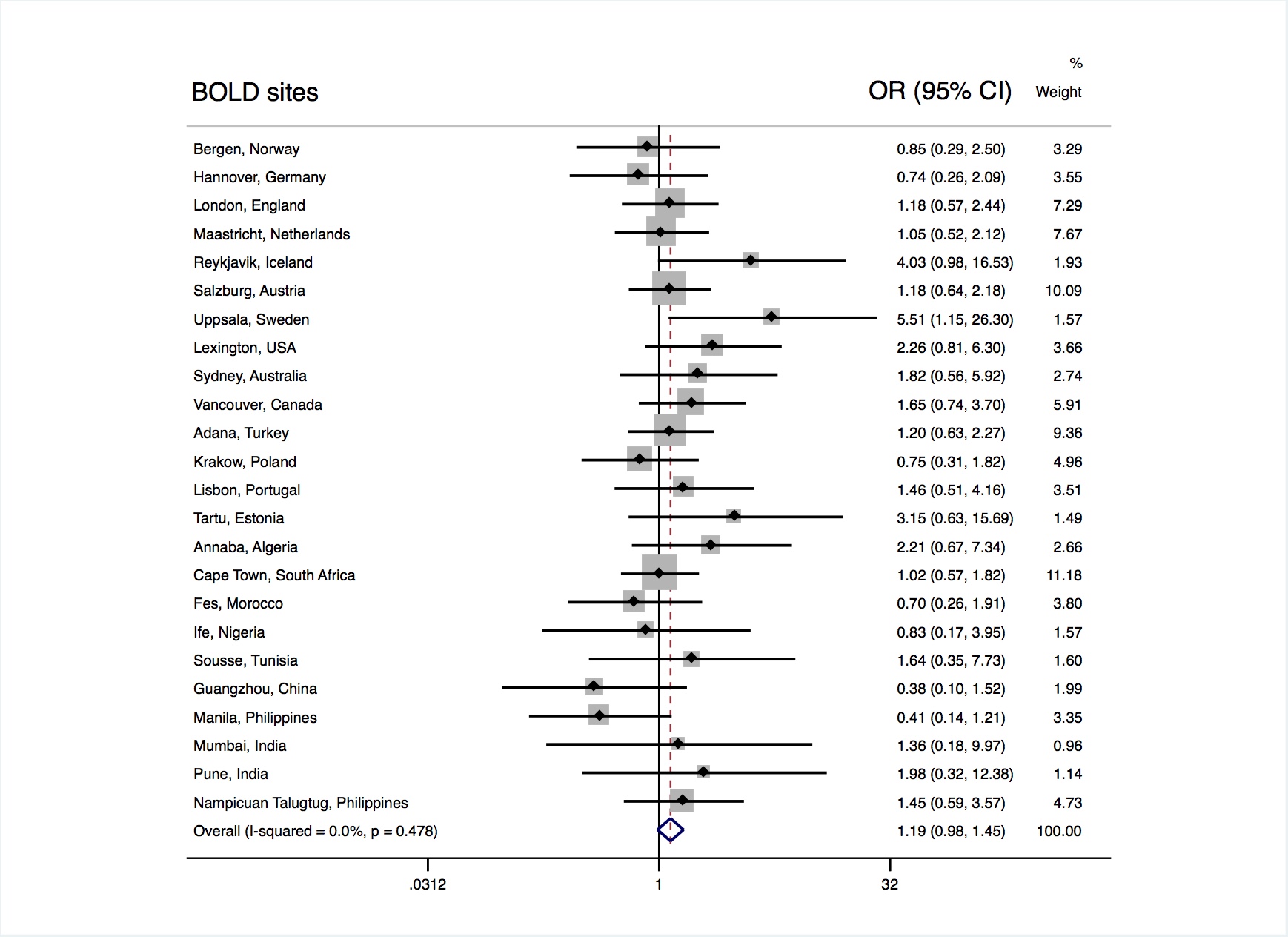

Supplement: Supplementary file 5 [file ERJ-00499-2017_Figure_S4.jpg]
